# A Stacking-Enhanced Support Vector Regression Model for Predicting the Hot Deformation Flow Stress of TC18 Alloy

**DOI:** 10.3390/ma19122615

**Published:** 2026-06-17

**Authors:** Xiang Jiang, Shuangxi Shi, Chenyang Lu, Xuan Shi, Shaoling Ding, Yaobiao Liang

**Affiliations:** 1 School of Mathematics and Statistics, Guilin University of Technology, Guilin 541006, China; 18570490501@163.com (X.J.);; 2 Institute of Collaborative Innovation, University of Macao, Macao 999078, China; 3 Guangxi Key Laboratory of Special Engineering Equipment and Control, Guilin University of Aerospace Technology, Guilin 541004, China; 4 Guangxi Region Precious Metal Materials Advanced Process Research Center, Guilin University of Aerospace Technology, Guilin 541004, China

**Keywords:** TC18 alloy, hot deformation behavior, support vector regression, stacking integrated algorithm, flow stress prediction

## Abstract

This study systematically investigates the hot deformation behavior of TC18 alloy under the conditions of deformation temperatures of 720–840 °C and strain rates of 0.001–1 s^−1^. Based on the stress–strain data obtained under the aforementioned process parameters, a support vector regression (SVR) model was established and further optimized by using a Stacking algorithm to enhance predictive accuracy. Although SVR and Stacking techniques have been applied previously in material constitutive modeling, this paper presents a systematic optimization framework specifically for TC18, integrating comprehensive experimental data, kernel selection, hyperparameter tuning, and Stacking-based model fusion. The polynomial kernel function was identified as optimal, and hyperparameters were tuned via grid search combined with five-fold cross-validation, which is determined as {*C* = 1000, *coef*0 = 1, *d* = 5, *ε* = 1, *γ* = 1}. The Stacking-SVR model exhibits significantly improved fitting and generalization performance compared to Poly-SVR, Arrhenius, XGBoost and MLP, with *RMSE*, *MAPE*, and *R*^2^ metrics of 2.7882, 0.0110, and 0.9973 on the training set, and 2.7956, 0.0169, and 0.9982 on the test set, respectively. Additionally, the proportion of samples with relative errors within 5% reaches 98.7% for the training set and 94.83% for the test set. These results indicate that the proposed framework not only possesses extremely high predictive accuracy, but also ensures strong generalization ability and interpretability in practical applications.

## 1. Introduction

### 1.1. Research Background

TC18 (Ti-5Al-5Mo-5V-1Fe-1Cr) titanium alloy is a lightweight and high-strength structural material and has gained extensive application in high-end equipment fields such as aerospace and marine engineering due to its excellent corrosion resistance, high-temperature stability, weldability, non-magnetic properties, and non-toxicity [1,2]. To meet the increasing demands for specific strength, specific stiffness and creep resistance, research on titanium alloys has been continuously advancing [3,4]. Among them, the α-β dual-phase titanium alloy has attracted significant attention for its superior high-temperature workability and capability for complex geometry forming [5]. As a typical representative of α-β dual-phase titanium alloys, TC18 alloy achieves a synergistic matching of α and β phases through precise control of key alloying elements such as aluminum, molybdenum, and vanadium [6], demonstrating excellent comprehensive mechanical properties and good adaptability to thermomechanical processing [7].

The microstructural characteristics of a material fundamentally determine its core mechanical properties, such as tensile strength, yield strength, ductility, and fatigue life, as well as the forming quality of the final product [8]. Thermomechanical processing and subsequent heat treatment are critical steps affecting the mechanical properties of high-strength titanium alloys. By precisely controlling parameters such as deformation temperature, strain rate, and deformation degree and optimizing the heat treatment process, effective microstructure refinement can be achieved, significantly enhancing the alloy’s comprehensive properties [9,10]. Notably, the hot deformation behavior of TC18 alloy is inherently complex and highly sensitive to processing parameters [11,12], and its flow stress is primarily governed by the coupled effects of deformation temperature, strain rate, and strain [13,14]. During conventional hot working processes such as forging and rolling, dynamic microstructural evolution occurs within the material, driven by the competitive interplay of mechanisms like work hardening, dynamic recovery, and dynamic recrystallization, which directly determines the macroscopic flow response of the alloy [15]. If the flow becomes unstable during the hot processing, this may result in severe defects such as coarse grains, an uneven microstructure, and excessive residual stress [16]. Therefore, systematically studying the evolution law of flow stress of TC18 alloy under different thermal processing conditions, and establishing an accurate constitutive model to describe its deformation behavior, has significant theoretical value and urgent engineering significance for optimizing the process route, reducing energy consumption and waste rate, etc.

In the prediction of the hot deformation behavior of metal materials, the traditional Arrhenius constitutive model has obvious limitations. Its hyperbolic sine function form is preset and has difficulty accurately describing the complex rheological behavior resulting from the coupling of multiple mechanisms such as work hardening and dynamic softening. On the other hand, the model parameters need to be calibrated one by one at different strain points and supplemented by polynomial fitting, which is a cumbersome process and prone to overfitting. Moreover, the model has limited ability to capture high-order nonlinear relationships among multiple variables. In these conditions, machine learning models, with their remarkable capabilities, can capture the complex nonlinear relationships among multiple variables. They have been widely applied in the constitutive relationship and microstructure modeling of titanium alloys [17]. Meanwhile, with the advancement of artificial intelligence technology, methods based on artificial intelligence have demonstrated unique advantages over traditional physical models [18,19]. They do not require prior knowledge of explicit mathematical expressions or underlying physical laws, nor do they rely on assumptions or manual data preprocessing to obtain outputs from experimental datasets [20]. These characteristics make them particularly suitable for modeling the thermoplastic flow behavior of titanium alloys [21].

### 1.2. Related Research

Among the existing literature, Guo et al. investigated the hot deformation behavior and mechanisms of the near-β titanium alloy TC18 in the single-phase region through isothermal compression tests. They predicted the flow behavior using a strain-compensated Arrhenius-type constitutive equation and found that instability predominantly occurred under high-strain-rate conditions [22]. Liu et al. developed a back-propagation (BP) neural network model to overcome the limitations of traditional methods, such as data sensitivity and strict model assumptions, thereby improving the prediction accuracy for the hot deformation behavior of near-β titanium alloys [23]. Shi et al. proposed a genetic-algorithm-optimized support vector regression (GA-SVR) model to predict the flow behavior of Ti-13Nb-13Zr alloy, achieving a stable average absolute relative error (AARE) below 0.18% [24]. Zhao et al. combined computer vision technology with a convolutional neural network (CNN) to construct a microstructure classification model for titanium alloys. By extracting feature maps from convolutional layers and fusing them with mechanical property data, a regression network is established. Furthermore, the two models are cascaded to enable direct prediction of the stress–strain curves [25]. Liu et al. utilized large-area stitching technology for gradient strain analysis of TC18 alloy and implemented an improved K-nearest neighbor algorithm optimized by back-propagation and particle swarm optimization (PSO-KNN). Their results demonstrated that the combination of high-throughput experiments and machine learning significantly enhanced the accuracy and efficiency of microstructure prediction [26].

Despite the significant progress made in the aforementioned studies, certain limitations persist. First, traditional constitutive models (e.g., Arrhenius-type equations) are based on phenomenological assumptions [27] and possess limited capability in describing the complex nonlinear responses induced by the coupled mechanisms of work hardening, dynamic recovery, and dynamic recrystallization during high-temperature deformation [28]. Second, physically based constitutive models (e.g., those based on dislocation density evolution) offer strong extrapolation capabilities, but their parameter calibration is complex, and accurately representing the underlying micromechanisms remains challenging [29]. Third, while existing single machine learning models (such as BP neural networks, SVR, GA-SVR) achieve high prediction accuracy under certain conditions, their generalization ability is often constrained by the distribution of training data, leading to insufficient stability across different combinations of hot working parameters and a tendency towards overfitting or underfitting. Finally, current research primarily focuses on comparative analysis between different modeling methods, with limited exploration of the systematic improvement effects of ensemble learning strategies on prediction performance, and a lack of in-depth investigation into multi-model fusion mechanisms and their application value in predicting the hot deformation of titanium alloys [30].

### 1.3. Research Motivation

To address the aforementioned limitations, this paper proposes an SVR-Stacking flow stress prediction model based on the Stacking ensemble learning framework. The support vector regression algorithm, grounded in statistical learning theory, has been widely applied in material prediction due to its robustness in high-dimensional feature spaces and its ability to effectively model nonlinear relationships among material-influencing factors [31,32,33]. In this study, SVR is selected as the base learner, allowing flexible selection of kernel functions based on input variable characteristics to identify the most suitable kernel function type for the given scenario, thereby enhancing prediction accuracy. Building upon this, the Stacking ensemble learning framework is introduced to integrate the prediction outputs from multiple base models. A meta-learner learns the optimal weight allocation for each base model, effectively improving the model’s robustness and generalization capability. Concurrently, grid search combined with cross-validation is employed for global optimization of model hyperparameters, overcoming the inherent limitations and instability of a single model under varying working conditions.

The major contributions of this study are as follows: (1) Constructing an SVR-Stacking ensemble learning model tailored for predicting the hot deformation flow behavior of TC18 alloy; (2) systematically investigating the influence of kernel function selection, hyperparameter configuration, and data scale on model performance; (3) demonstrating through comparative experiments that the Stacking-SVR model significantly outperforms the other models in both fitting accuracy and generalization capability; (4) providing an efficient, stable, and practically valuable predictive tool for optimizing the hot working process of titanium alloys.

## 2. Experimental Procedure and Methodology

### 2.1. Experimental Procedure

**Figure 1:**
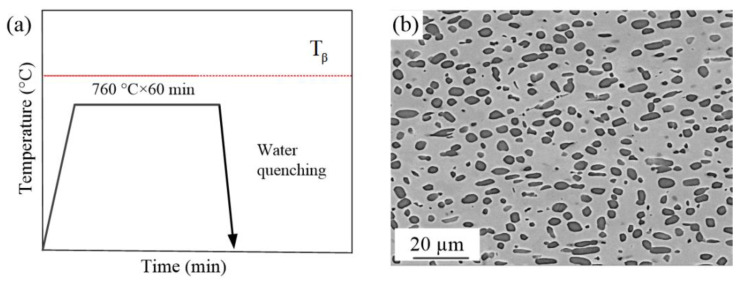
Heat treatment process (**a**) and equiaxed microstructure (**b**) of TC18 alloy.

The raw material used in this study is a forged TC18 titanium alloy rod, with its nominal chemical composition (mass fraction) presented in Table 1. The phase transformation temperature of the alloy was determined to be approximately 1113 ± 5 K by using the metallographic method. Prior to the hot compression experiments, the forged TC18 alloy rod was subjected to an isothermal holding at 760 °C for 60 min, followed by water quenching, in order to obtain a uniform equiaxed microstructure, as shown in Figure 1. The resulting equiaxed α phase is homogeneously distributed within the β matrix, exhibiting a volume fraction of approximately 26.94% and an average grain size of about 4.0 μm.

Cylindrical specimens with dimensions of Φ10 mm × 15 mm were extracted from the heat-treated bulk alloy via wire electrical discharge machining and subsequently mechanically polished to remove surface imperfections. Isothermal hot compression experiments were performed on a Gleeble 3800 thermo-mechanical simulator (manufactured by Dynamic Systems Inc., Poestenkill, NY, USA) at deformation temperatures of 720, 760, 800, and 840 °C and strain rates of 0.001, 0.01, 0.1 and 1 s^−1^, respectively [34]. All experiments were conducted under an argon protective atmosphere to mitigate surface oxidation. To further minimize frictional effects between the specimen and the anvils, thin graphite and tantalum foils were interposed during compression [35].

The support vector regression algorithm, grounded in statistical learning theory, has been widely applied in material prediction due to its robustness in high-dimensional feature spaces and its ability to effectively model nonlinear relationships among material-influencing factors. The support vector regression algorithm, grounded in statistical learning theory, has been widely applied in material prediction due to its robustness in high-dimensional feature spaces and its ability to effectively model nonlinear relationships among material-influencing factors.

The detailed experimental procedure was conducted as follows. First, the specimens were heated to the target temperature at a heating rate of 10 °C/s and held for 180 s to ensure uniform temperature distribution throughout the material. Subsequently, all specimens underwent compression to a height reduction of 60%, corresponding to a true strain of 0.9. Immediately after deformation, the specimens were quenched in water, while the testing system continuously recorded the true stress–true strain curves throughout the deformation process. In total, 288 sets of valid true stress–strain data were obtained (4 temperatures × 4 strain rates × 18 strain points ranging from 0.05 to 0.9 in intervals of 0.05) [36,37], providing a comprehensive and reliable dataset for subsequent machine learning modeling and analysis [38].

To ensure the reliability and reproducibility of the compression experiments, each test under a specific temperature and strain rate was repeated at least three times. The resulting true stress–strain curves were compared, and the averaged values were used for machine learning model training. The standard deviation of peak stresses across repeated tests was maintained within ±2–3%, indicating low experimental variability and confirming that the dataset offers a robust and consistent foundation for model development.

### 2.2. Model Methods

#### 2.2.1. Construction of the SVR Constitutive Model

**Figure 2:**
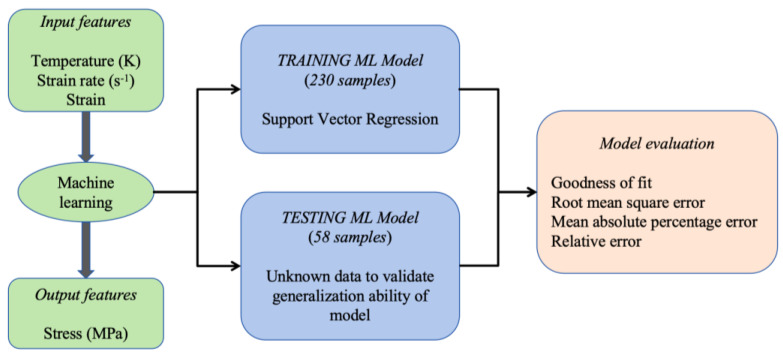
Framework of the achine learning model.

Machine learning models have been extensively employed in the investigation of thermoplastic deformation behaviors of alloys due to their strong generalization capabilities, enabling the extraction of complex patterns from input data and accurate prediction of material responses [39]. In this study, the input variables comprise true strain, deformation temperature, and strain rate, while the output variable is flow stress; these parameters are critical in governing the hot workability of the alloy [40,41]. A total of 288 experimental datasets were obtained, of which 80% (approximately 230 sets) were randomly allocated as the training set for model training, and the remaining 20% (approximately 58 sets) were reserved as the test set to evaluate the model’s predictive performance on unseen data. Figure 2 illustrates the overall framework of the machine learning-based constitutive model.

Among various machine learning approaches, the support vector regression (SVR) model has been extensively applied to regression problems owing to its superior predictive performance and robust generalization capability, making it highly favored by researchers. The fundamental principle of SVR is to minimize the prediction error while simultaneously controlling model complexity, and its basic form is shown in Equation (1) [42]:

(1) $$f\left(x\right)=\omega \cdot \phi \left(x\right)+b$$

In Equation (1), ω is the multi-dimensional column vector, b is the intercept term, and $\phi \left(x\right)$ is the feature mapping function that projects the input space to a high-dimensional feature space.

Assume the original data is $\left(x_{1},y_{1}\right),\left(x_{2},y_{2}\right),\left(x_{3},y_{3}\right),\cdots ,\left(x_{i},y_{i}\right),\cdots ,\left(x_{k},y_{k}\right),\;\mathrm{where}\;x_{i},y_{i}\in R$, and the function $f\left(x\right)$ aims to approximate all data points. The optimal regression function is determined by solving the following optimization problem:

(2) $$min\frac{1}{2}{\left\|\omega \right\|}^{2}+C\sum_{i=1}^{n}\left(\xi_{i}+\xi_{i}^{*}\right)$$

(3) $$s.t.\left\{\begin{array}{c} y_{i}-\omega \cdot \phi \left(x_{i}\right)-b\leq \varepsilon +\xi_{i}\\ \omega \cdot \phi \left(x_{i}\right)+b-y_{i}\leq \varepsilon +\xi_{i}^{*}\\ {\;\xi }_{i},\xi_{i}^{*}\geq 0 \end{array}\right.$$

where Equation (2) represents the objective function; $\xi_{i}$ and $\xi_{i}^{*}$ are the relaxation variables that affect the regression accuracy; C is the regularization parameter controlling the trade-off between model complexity and training error; ε is an insensitive loss parameter that has a significant impact on the regression accuracy. Equation (3) defines the model constraints, and n is the number of samples.

By introducing Lagrange multipliers $\alpha_{i}\;\mathrm{and}\;\alpha_{i}^{*}$, the primal problem is transformed into its dual form:

(4) $$max\left[-\frac{1}{2}\sum_{i,j=1}^{n}\left(\alpha_{i}-\alpha_{i}^{*}\right)\left(\alpha_{j}-\alpha_{j}^{*}\right)K\left(x_{i},x_{j}\right)-\varepsilon \sum_{i=1}^{n}\left(\alpha_{i}-\alpha_{i}^{*}\right)+\sum_{i=1}^{n}y_{i}\left(\alpha_{i}-\alpha_{i}^{*}\right)\right]$$

(5) $$s.t.\left\{\begin{array}{c} \sum_{i=1}^{n}\left(\alpha_{i}-\alpha_{i}^{*}\right)=0\\ \&0\leq \alpha_{i},\alpha_{i}^{*}\leq C \end{array}\right.$$

where $K\left(x_{i},\;x\right)={\phi \left(x_{i}\right)}^{T}\phi \left(x_{j}\right)$ is the kernel function. The optimal solution of the SVR model can then be obtained. Ultimately, the prediction function is expressed as

(6) $$f\left(x\right)=\sum_{i=1}^{n}\left(\alpha_{i}^{*}-\alpha_{i}\right)K\left(x_{i},x\right)+e$$

#### 2.2.2. Grid Search Method

Grid search is a classical and widely adopted hyperparameter optimization strategy in machine learning. Its fundamental principle is to discretize the hyperparameter space of a model into a structured multi-dimensional grid and systematically evaluate the performance of all possible hyperparameter combinations within that grid to identify the configuration that maximizes the model’s generalization capability. Conceptually, grid search represents a form of global exhaustive optimization and is particularly well-suited for the five key hyperparameters in this study, given their low dimensionality and clearly defined search ranges.

Nevertheless, this approach exhibits certain limitations. Random partitioning of the dataset for validation may introduce bias in performance estimation and result in suboptimal data utilization. Additionally, grid search does not inherently quantify the stability of selected hyperparameters nor provide an assessment of model robustness. Consequently, in practical applications, grid search is often integrated with cross-validation techniques to mitigate these shortcomings and ensure more reliable hyperparameter selection.

#### 2.2.3. Cross-Validation

**Figure 3:**
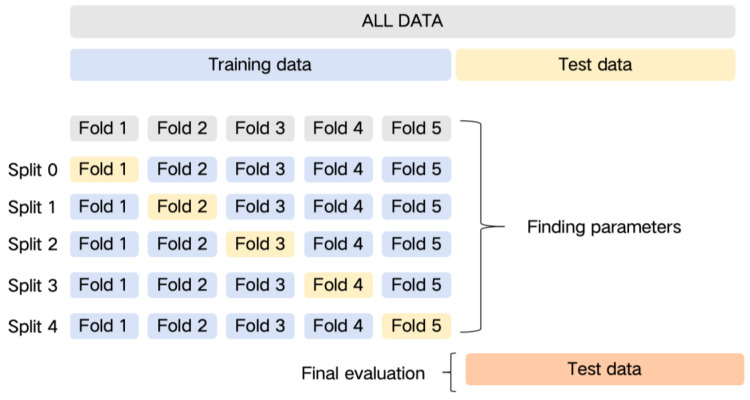
Schematic diagram of five-fold cross-validation.

Cross-validation facilitates low-bias and low-variance estimation of a model’s true generalization error by systematically partitioning the dataset, performing iterative training, and aggregating performance metrics across multiple rounds on labeled data, and its schematic illustration is presented in Figure 3. This approach effectively mitigates the random bias associated with a single data split, thereby addressing the inherent limitations of grid search [43]. Consequently, the integration of grid search with cross-validation enhances both the accuracy and efficiency of hyperparameter optimization, ensuring the robustness of model generalization and the statistical reliability of the resulting optimal parameters. The number of folds (K value) in cross-validation directly influences the trade-off between model bias and variance: a low K value can result in high bias in performance estimation, whereas a high K value may increase variance.

#### 2.2.4. Stacking Ensemble Algorithm

**Figure 4:**
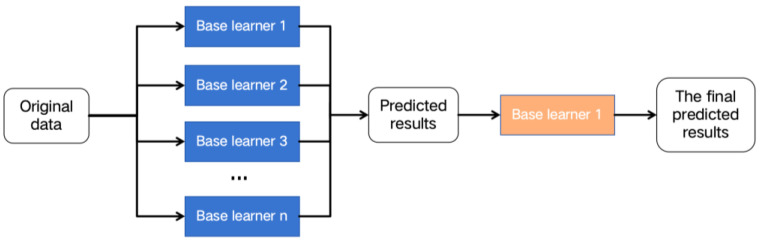
Flowchart of the Stacking algorithm.

The Stacking ensemble algorithm is a hierarchical ensemble algorithm. As an advanced hierarchical integration strategy, its core principle lies in achieving collaborative optimization among heterogeneous models through a multi-stage learning mechanism, thereby mitigating the limitations of individual algorithms when dealing with complex data distributions [44]. The algorithm initially employs multiple heterogeneous base learners to form a first-layer parallel learning structure. Subsequently, the predictions generated by the base learners are treated as secondary features to train a higher-level meta-learner, yielding a complete ensemble framework with improved predictive accuracy and stronger generalization capability [45]. Figure 4 is the flowchart of Stacking algorithm.

In this study, the proposed Stacking-SVR framework comprises three hierarchical layers. The first layer is the base learner layer, where heterogeneous models are independently trained using deformation temperature, strain rate, and strain as input features, while flow stress is used as the target output. The base learner layer consists of a polynomial kernel SVR model (Poly-SVR) and a random forest regression model, because Poly-SVR performed best in this study and RF can enhance the diversity. In order to prevent information leakage, we used five-fold cross-validation to generate out-of-fold predictions from each base learner during training.

The second layer functions as a feature reconstruction layer. For each training sample, the predictions of the base learners are treated as meta-feature vectors. The meta-feature vectors from Poly-SVR and the random forest model are horizontally concatenated to form a new feature matrix, with dimensionality corresponding to the number of base learners. This reconstructed feature matrix effectively integrates complementary information from the base models and provides reliable input for the meta-model layer.

The third layer functions as the meta-model layer, in which a linear regression model is employed as the meta-learner to perform secondary training on the reconstructed meta-features. The linear regression meta-learner offers several advantages: (1) it allows quantification of the contribution of each base learner via regression coefficients, providing interpretability of the fusion mechanism; (2) L2 regularization suppresses multicollinearity among meta-features, improving model stability; and (3) the linear mapping preserves complementary information from the base learners while avoiding unnecessary nonlinear noise, ensuring robust and reliable predictions. Furthermore, the hyperparameters of the meta-learner were optimized using grid search in combination with cross-validation to enhance generalization capability and prevent overfitting [46].

#### 2.2.5. Evaluation Index

To evaluate the predictive performance of the models, this study selects root mean square error (*RMSE*), mean absolute percentage error (*MAPE*) and coefficient of determination (*R*^2^) as evaluation metrics. These metrics provide complementary perspectives on model performance, capturing absolute error, relative error percentage, and model explanatory power, respectively. *RMSE* is defined as the square root of the mean of the squared differences between the predicted and actual values; *MAPE* quantifies the average absolute error as a percentage of the actual values, providing a normalized measure of relative deviation; R^2^ represents the proportion of the variance in the target variable explained by the model, serving as an indicator of goodness of fit. Lower values of *RMSE* and *MAPE* correspond to smaller fitting deviations, whereas higher *R*^2^ values indicate better model fit. The calculation formulas for these three evaluation metrics are as follows, respectively:

(7) $$\mathrm{RMSE}=\sqrt{\frac{1}{n}\sum_{i=1}^{n}{\left(Y_{i}-{\hat{Y}}_{i}\right)}^{2}}$$

(8) $$\mathrm{MAPE}=\frac{100\%}{n}\sum_{i=1}^{n}\left|\frac{Y_{i}-{\hat{Y}}_{i}}{Y_{i}}\right|$$

(9) $$R^{2}=1-\frac{\sum_{i=1}^{n}{\left(Y_{i}-{\hat{Y}}_{i}\right)}^{2}}{\sum_{i=1}^{n}{\left(Y_{i}-\overset{-}{Y}\right)}^{2}}$$

In these equations, ${\hat{Y}}_{i}$ is the predicted stress, $Y_{i}$ is the experimental stress data, $\overset{-}{Y}$ is the mean of the predicted stress data, and *n* is the number of samples.

### 2.3. Comparison Models

#### 2.3.1. Arrhenius Model

In order to compare the advantages of machine learning models in describing the thermal deformation behavior of metal materials, we selected a classic traditional constitutive model, Arrhenius, to describe the nonlinear relationship between the rheological stress and the deformation temperature, strain rate, and strain. Arrhenius is based on the theory of thermal activation of dislocation motion, and its basic form is

(10) $$\dot{\varepsilon }=A{\left[sinh(\alpha \sigma )\right]}^{n}exp(-Q/RT)$$

We introduce the Zener–Hollomon parameter Z, which enables us to inversely solve for the rheological stress σ:

(11) $$Z=\dot{\varepsilon }exp(-Q/RT)$$

(12) $$\sigma =\frac{1}{\alpha }ln\{{\left(Z/A\right)}^{1/n}+{\left[{\left(Z/A\right)}^{2/n}+1\right]}^{1/2}\}$$

The activation energy Q represents the energy barrier that must be overcome for atomic migration during deformation. When Q is comparable to the self-diffusion activation energy of the alloy, the deformation is primarily governed by steady-state creep controlled by dislocation motion. Conversely, a significantly higher Q value indicates the involvement of additional energy dissipation mechanisms, such as second-phase particle pinning or dynamic precipitation. The stress exponent n can be employed to identify the dominant deformation mechanism. Values of n exceeding 5 are often associated with processes such as dynamic recrystallization or the detachment of dislocations from second-phase particles under high-stress conditions [47].

The parameter α serves to unify the constitutive expressions across both low- and high-stress regimes. Since the model constants vary with strain, they are determined at multiple discrete strain points. Subsequently, these strain-dependent constants are fitted using a fifth-degree polynomial (i.e., strain compensation), and the resulting expressions are incorporated into the constitutive equation to predict the flow stress at any arbitrary strain.

#### 2.3.2. XGBoost Model

The sample size of the data obtained in this experiment is small; therefore, we selected another gradient boosting method for comparison. XGBoost model (Extreme Gradient Boosting) is an efficient ensemble learning method based on gradient boosting decision trees (GBDT). The core idea is to iteratively train a series of regression trees, with each tree fitting the residuals of the previous model, thereby gradually reducing the prediction error. The model prediction can be expressed as

(13) $${\hat{y}}_{i}=\sum_{k=1}^{K}f_{k}(x_{i}),\;f_{k}\in F$$

where ${\hat{y}}_{i}$ represents the predicted value of the i-th sample, $x_{i}$ is the input feature vector, F is the function space of the regression tree, and K is the number of trees. The objective function of XGBoost is defined as

(14) $$OBj=\sum_{i=1}^{n}l(y_{i},{\hat{y}}_{i})+\sum_{k=1}^{K}\Omega (f_{k})$$

where $l(y_{i},{\hat{y}}_{i})$ represents the loss function and $\Omega (f)=\gamma T+\frac{1}{2}\lambda \sum_{j=1}^{T}\omega_{j}^{2}$ is the regularization term for the tree, used to control the number of leaf nodes T and the weights of leaf nodes $\omega_{j}$, thereby reducing the model complexity and preventing overfitting.

XGBoost approximates the objective function through the second-order Taylor expansion for efficient optimization of gradients and incremental tree growth. The algorithm supports column sampling, parallel computing, and handling of missing values, making it highly stable in nonlinear and small-sample data scenarios. In this study, the input features are deformation temperature, strain rate, and true strain. By iteratively fitting residuals, XGBoost can capture the complex nonlinear relationships between these features and the flow stress, thereby providing high accuracy and strong robustness for predictions.

#### 2.3.3. MLP

Multi-Layer Perceptron (MLP) is a typical feedforward artificial neural network that achieves complex relationship fitting from input features to output targets through multiple layers of nonlinear mapping. For a sample $x_{i}={\left[x_{1},x_{2},\ldots ,x_{d}\right]}^{T}$, the predicted output of MLP can be expressed as

(15) $${\hat{y}}_{i}=f_{L}(W_{L}f_{L-1}(\ldots f_{2}(W_{2}f_{1}(W_{1}x_{i}+b_{1})+b_{2})\ldots )+b_{L})$$

where L is the number of network layers, $W_{L}$ and $b_{L}$ are the weight matrix and bias vector of the L-th layer, respectively, and $f_{L}(\cdot )$ is the activation function.

The model training employs the back-propagation algorithm combined with an optimizer (in this paper, Adam is selected). It iteratively updates the network parameters to gradually reduce the prediction error. The MLP can effectively capture the nonlinear relationships among various variables through regularization terms and EarlyStopping strategies, thereby achieving high-precision predictions and enhancing the generalization ability of the model.

## 3. Results

### 3.1. Flow Curves

**Figure 5:**
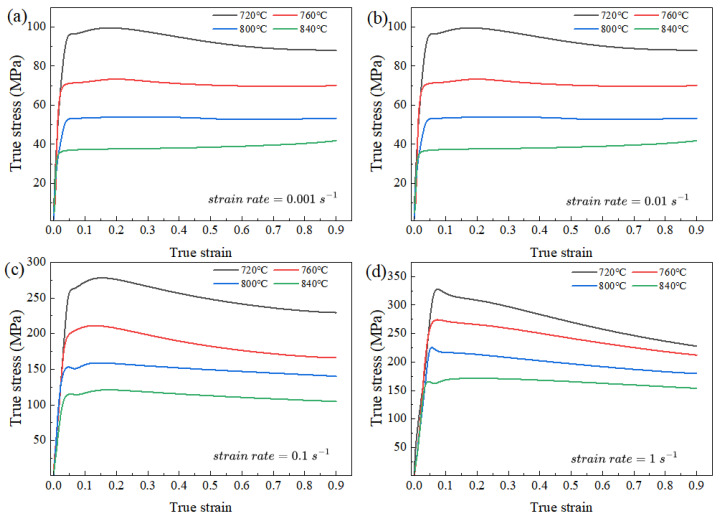
Flow curves of TC18 alloy under different deformation conditions: (**a**) 0.001 s^−1^; (**b**) 0.01 s^−1^; (**c**) 0.1 s^−1^; (**d**) 1 s^−1^.

Figure 5 shows the stress–strain curves of TC18 alloy tested under different deformation temperatures (720, 760, 800 and 840 °C) and strain rates (0.001, 0.01, 0.1 and 1 s^−1^). As shown, the flow stress exhibits a strong dependence on both deformation temperature and strain rate. The flow stress is negatively correlated with temperature, decreasing as the temperature rises, and is positively correlated with strain rate, increasing as the strain rate increases when another variable is fixed. Under all deformation conditions, the flow curves display a consistent evolution pattern. As true strain increases, the TC18 alloy sequentially undergoes three stages: work hardening, dynamic softening, and dynamic equilibrium. In the initial stage of deformation, rapid dislocation proliferation dominates the work hardening process, leading to a sharp increase in stress. Subsequently, the dynamic softening effect gradually strengthens and counteracts work hardening. Finally, work hardening and dynamic softening reach a dynamic equilibrium, and the stress tends to stabilize. This phenomenon is commonly observed in alloys and stems from the fact that the internal microstructure evolution during hot deformation of metallic materials follows similar physical metallurgy principles—specifically, processes such as dislocation motion, grain boundary migration, and energy transfer share analogous physical mechanisms. However, due to variations in crystal structure, alloying element content, and second-phase particle distribution among different materials, the specific characteristics of their deformation behavior exhibit subtle differences [48].

**Figure 6:**
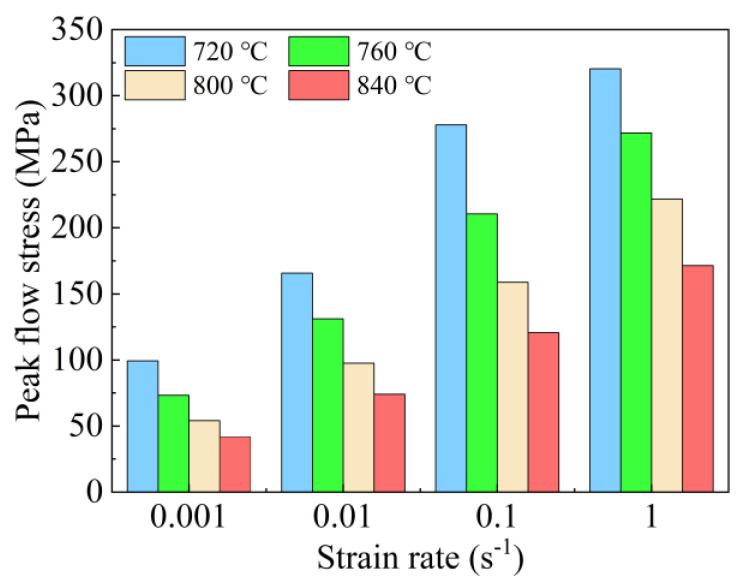
Influence of deformation conditions on peak stress.

The peak flow stress changes in TC18 alloy under different deformation conditions are shown in Figure 6. Under the condition of 720 °C and 1 s^−1^, the flow stress reaches its maximum value of 327.80 MPa. When the temperature increases to 840 °C and the strain rate decreases to 0.001 s^−1^, the stress drops to 41.83 MPa, representing a difference of 285.97 MPa. This indicates that deformation temperature and strain rate have a significant regulatory effect on flow stress. The underlying mechanism can be explained as follows. At a high strain rate (e.g., 1 s^−1^), the shortened deformation time causes the dislocation proliferation rate to exceed the annihilation rate, leading to the accumulation of dislocation density. This enhances the material’s resistance to deformation and increases the flow stress. As the temperature rises, the stress exhibits a decreasing trend. This is mainly attributed to two aspects: on the one hand, the increase in atomic kinetic energy reduces the resistance to dislocation motion; on the other hand, when the temperature approaches the β-transus point, the TC18 alloy transforms from the α phase (hexagonal close-packed structure, 3 slip systems) to the β phase (body-centered cubic structure, 12 slip systems), and the increase in the number of slip systems significantly reduces the deformation resistance. Furthermore, at a low strain rate (e.g., 0.001 s^−1^), the extended deformation time not only facilitates dislocation rearrangement and annihilation but also fully activates softening mechanisms such as dynamic recovery (DRV) and dynamic recrystallization (DRX), thereby further reducing the stress required for deformation. This fully confirms the regulatory effect of strain rate on flow stress [49].

### 3.2. SVR Model

#### 3.2.1. Determination of the Kernel Function

In the solution process of SVR, the data is implicitly mapped to a high-dimensional space through the kernel function $K\left(x_{i},x_{j}\right)={\phi \left(x_{i}\right)}^{T}\phi \left(x_{j}\right)$ to solve the nonlinear regression problem. Here, ϕ(x) is the function that maps the data to a high-dimensional space, and the kernel function $K\left(x_{i},x_{j}\right)$ directly computes the inner product after mapping. In this work, four typical kernel functions were evaluated, and their characteristics are summarized in Table 2.

**Figure 7:**
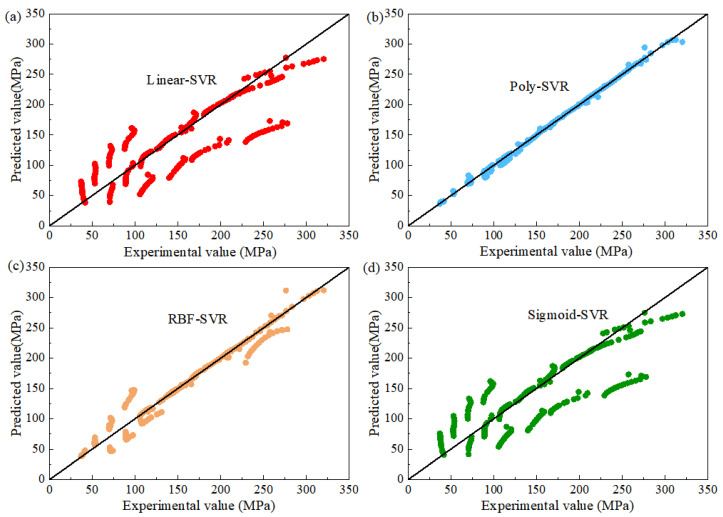
Regression analysis results of different kernel functions on the training set: (**a**) Linear; (**b**) Poly; (**c**) RBF; (**d**) Sigmoid.

For the nonlinear flow stress behavior of TC18 alloy, selecting an appropriate kernel function is essential to balance fitting accuracy with generalization performance. The regression analysis results of the training sets for SVR model with four different kernel functions are shown in Figure 7. When the polynomial kernel function is utilized, the predicted flow stress values show the closest agreement with experimental measurements. The data distribution characteristics are as follows: the vast majority of data points are closely distributed around the optimal fitting line, with only a few showing slight deviations, as illustrated in Figure 7b. In contrast, for the SVR models constructed using the linear, RBF and Sigmoid kernel functions, the training-set data points deviate significantly from the optimal fitting line, and the degree of dispersion is notably higher than that of the polynomial kernel model, as clearly evidenced by the visualization results.

**Figure 8:**
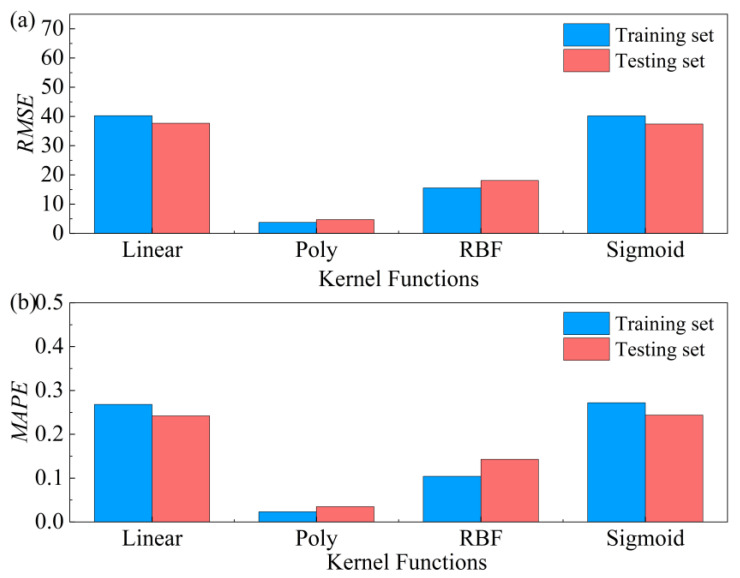
RMSE and MAPE of different kernel functions: (**a**) *RMSE*; (**b**) *MAPE*.

To further compare the fitting performance of different SVR models, this work employs three evaluation metrics: *RMSE*, *MAPE* and *R*^2^. Figure 8 presents the calculated *RMSE* (Figure 8a) and *MAPE* (Figure 8b) results for SVR models with different kernel functions. The results indicate that the fitting performance of the different kernel functions, in descending order, is as follows: polynomial kernel function, RBF kernel function, Sigmoid kernel function and linear kernel function. Among them, the linear kernel function exhibits the worst fitting performance, with an *RMSE* of 40.2789 on the training set and 37.6952 on the test set, and *MAPE* values exceeding 0.24 for both datasets. On the contrary, the polynomial kernel function achieves the best performance in terms of both *RMSE* and *MAPE*: it yields the lowest *RMSE* on the training set (3.7746) and the lowest *RMSE* on the test set (4.6864); meanwhile, its *MAPE* values on the training and test sets are also the smallest, reaching 0.0233 and 0.0351, respectively.

**Figure 9:**
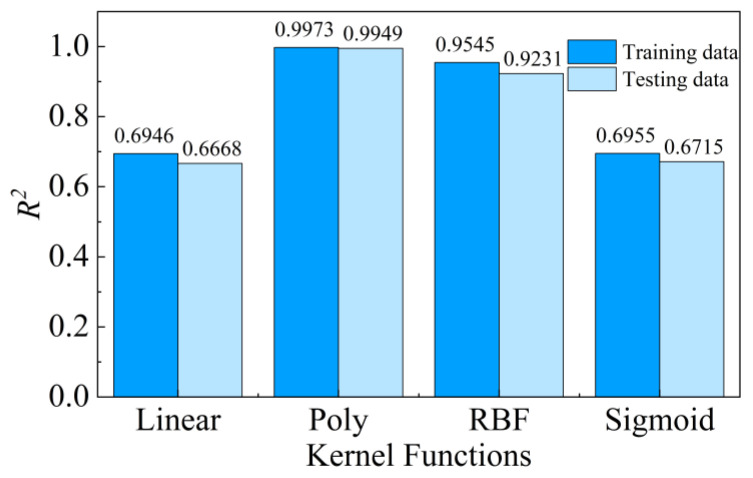
*R*^2^ values of SVR models with different kernel functions on the training and test sets.

Figure 9 presents the *R*^2^ of the SVR models with four different kernel functions. As shown in Figure 9, the polynomial kernel function still performs best with a value of 0.9973 for the training set and 0.9949 for the test set. In contrast, when the linear kernel function or the Sigmoid kernel function is selected, the fitting performance for both the training and test sets is unsatisfactory because their *R*^2^ values fall below 0.7 in both cases. Therefore, in this study, the SVR model should adopt the polynomial kernel function, which enables the model to achieve the optimal fitting performance.

#### 3.2.2. Hyperparameter Optimization

When we choose Poly-SVR to predict the flow stress of TC18 alloy, the model’s fitting performance is primarily determined by five key hyperparameters. To ensure a rational configuration, the value ranges and regulatory effects of these hyperparameters were determined by comprehensively considering theoretical principles, the characteristics of the thermal deformation data, and the mainstream parameter optimization intervals reported in related research on machine learning in the materials field. This avoids underfitting or overfitting cases that may be caused by extreme parameter values. The specific details of the parameters are shown in Table 3.

To optimize the hyperparameters of the SVR model, a grid search method combined with cross-validation was employed. During cross-validation, we used the mean square error (*MSE*) to evaluate model performance, and the optimal hyperparameter configuration is identified as the combination that minimizes the *MSE* within the defined parameter space. Equation (16) is the calculation formula for *MSE*:

(16) $$\delta_{MSE}=\frac{1}{n}\sum_{i=1}^{n}{\left(y_{i}-{\hat{y}}_{i}\right)}^{2}$$

In the formula, $y_{i}$ represents the true value, ${\hat{y}}_{i}$ is the predicted value of the model, and *n* is the number of samples in the test set. The five hyperparameters of the SVR model were C, coef0, d, ε and γ, and the numbers of candidate values were 9, 3, 4, 5 and 6, respectively, resulting in a total of 3240 hyperparameter combinations. The top best 10 *MSE* values calculated for each validation subset under each combination are summarized in Table 4.

Table 4 summarizes the fitting performance of the SVR model under different hyperparameter combinations. The labels split 0 to split 4 represent the first to fifth mutually exclusive subsets generated from the original dataset during five-fold cross-validation respectively. When the hyperparameters are configured as {C=1000, coef0=1, d=5, ε=1, γ=1}, the SVR model achieves the best fitting performance, with an average *MSE* of 33.28 across the five validation subsets and ranking first among all 3240 hyperparameter combinations. Therefore, for the polynomial kernel function, this hyperparameter combination can be considered the optimal configuration.

#### 3.2.3. Effect of Training-Set Proportion

During the construction of the SVR model, the proportion of training data plays a critical role in determining both fitting accuracy and predictive performance. To systematically investigate the influence of dataset size on model robustness and generalization capability, a random sampling strategy was employed. The proportion of the training set was progressively increased from 50% to 90% in increments of 10%, resulting in five data partitioning schemes in which 50%, 60%, 70%, 80%, and 90% of the original dataset were allocated for model training, respectively. For each partition, *RMSE*, *MAPE*, and *R*^2^ values were calculated to evaluate fitting accuracy and predictive stability.

**Figure 10:**
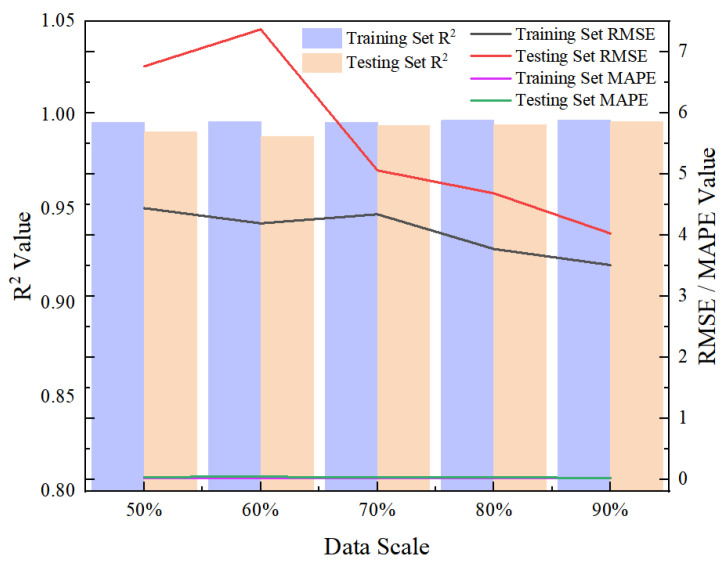
Influence of different training-set scales on model results.

As illustrated in Figure 10, increasing the proportion of training data generally enhances model performance. Larger training datasets enable the SVR model to more effectively capture the nonlinear relationships among true strain, deformation temperature, strain rate, and flow stress. Consequently, both *RMSE* and *MAPE* for the training and test sets decrease, while *R*^2^ increases, indicating improved learning capability and reduced prediction error.

However, the selection of the training-set ratio should not only focus on the size of the evaluation indicators, but also achieve an appropriate balance between the model’s learning ability and the reliable assessment of its generalization performance. An excessively large training set will reduce the number of independent test samples, which may lead to an overly optimistic estimation of the predictive performance and a decrease in the statistical reliability of the generalization evaluation. On the contrary, an overly small training set may limit the model’s ability to learn the hidden complex nonlinear relationships in the data.

Taking these factors into consideration, we chose the most common sample set division ratio in machine learning. We allocated 80% of the dataset for training and the remaining 20% for testing. This division strategy provides sufficient data for learning the potential deformation behavior, while also retaining enough independent samples for reliable model evaluation. Despite the dataset size being relatively limited, the SVR model consistently exhibits low prediction errors and stable *R*^2^ values across different training-set proportions, demonstrating strong predictive capability and robust generalization performance without significant overfitting. The stable performance observed on the test set further indicates that the model actually captures the intrinsic nonlinear deformation behavior of the alloy rather than merely memorizing the training data. Nevertheless, the absence of an independent external validation dataset remains a limitation of the present study. Future work will therefore focus on validating the proposed framework using additional experimental datasets under a broader range of deformation conditions.

### 3.3. Optimization of the SVR Model Using the Stacking Ensemble Algorithm

#### 3.3.1. Evaluation of Model Fitting and Generalization

**Figure 11:**
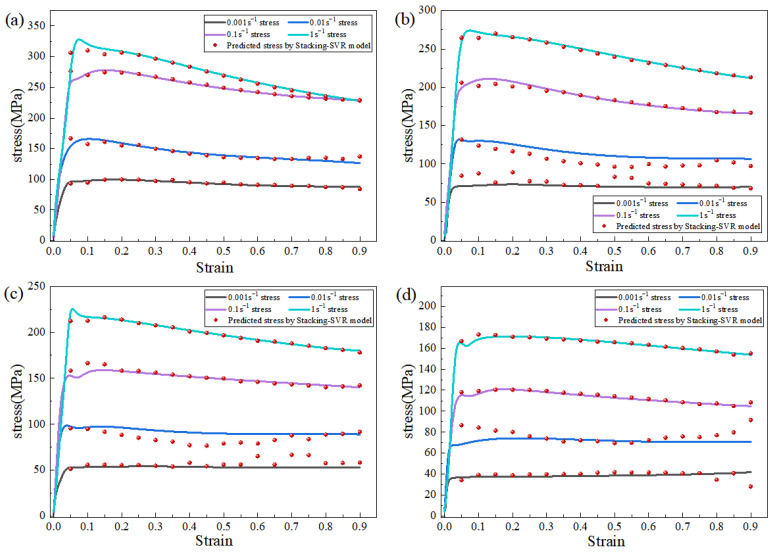
Comparison between the experimental stress and stress predicted by Stacking-SVR model at deformation conditions: (**a**) 720 °C; (**b**) 760 °C; (**c**) 800 °C (**d**) 840 °C.

Figure 11 compares the experimental true stress–true strain curves of TC18 alloy with the corresponding stresses predicted by the Stacking-SVR model under different deformation conditions. It is seen that the predicted values exhibit excellent agreement with the experimental data, indicating that the proposed model can effectively capture the nonlinear flow behavior of TC18 alloy during hot deformation. In particular, the model successfully reproduces the key characteristics of the flow curves, including the rapid stress increase during the initial deformation stage, the subsequent peak or near-steady-state response, and the gradual softening behavior at larger strains.

As shown in Figure 11a–d, the predicted stress remains generally consistent with the experimental curves across different deformation temperatures and strain rates, indicating the strong fitting accuracy and generalization capability of the Stacking-SVR model. Some local deviations can still be observed, particularly during the early stage of deformation and under certain low or intermediate strain-rate conditions. These discrepancies may be attributed to the limited size of the experimental dataset as well as the complex coupling effects among work hardening, dynamic recovery, and dynamic recrystallization mechanisms.

**Figure 12:**
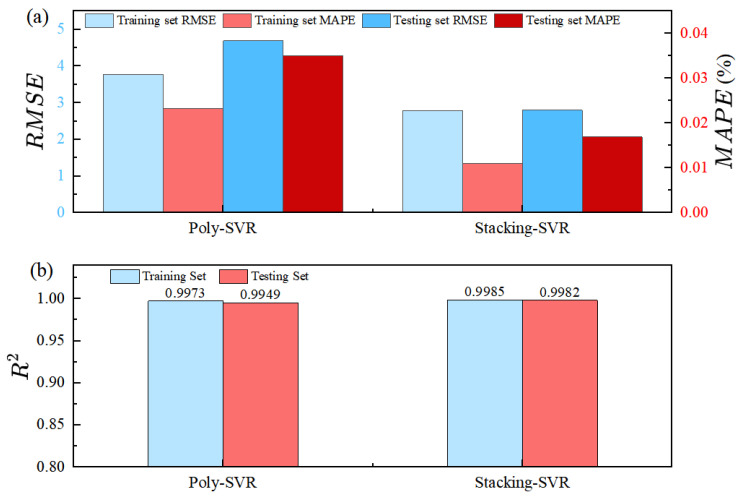
Comparison of the fitting effect of the Stacking-SVR and Poly-SVR: (**a**) *RMSE* and *MAPE*; (**b**) *R*^2^.

Figure 12 presents the predictive results of the SVR model after optimization using the Stacking ensemble algorithm. Compared with the SVR model before optimization, the Stacking-SVR model demonstrates markedly improved fitting performance. For the training set, the *RMSE* of Stacking-SVR is only 2.7882, which is lower than the 3.7746 of Poly-SVR, representing a reduction of 26.13%; the *MAPE* of Stacking-SVR is 0.0110, a decrease of 52.79% compared with Poly-SVR. The *R*^2^ of the Stacking-SVR training set is 0.9973, surpassing that of the original SVR model. These results indicate that the Stacking-optimized model indeed exhibits superior fitting performance in the training set.

Evaluation of model prediction and generalization on the test set further confirms the advantages of Stacking-SVR. The *RMSE* and *MAPE* of Stacking-SVR on the test set are 2.7956 and 0.0169 respectively, both substantially lower than those of Poly-SVR. Moreover, the *R*^2^ of the Stacking-SVR test set reaches 0.9982, exceeding the 0.9949 achieved by the original model. These findings demonstrate that the Stacking-optimized model achieves a significant improvement in generalization capability.

Furthermore, the concept of relative error was introduced to quantify the magnitude of prediction error relative to the overall scale of the measured quantity. Smaller relative error values indicate better model fitting performance. The calculation formula is provided as Equation (17):

(17) $$\eta =\frac{\left|y_{i}-{\hat{y}}_{i}\right|}{y_{i}}\times 100\%$$

**Figure 13:**
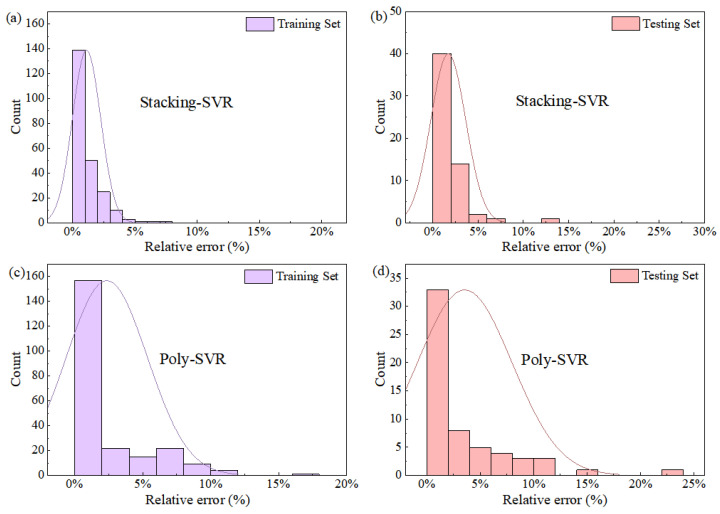
Relative error distributions of the developed machine learning models: (**a**) training set of Stacking-SVR; (**b**) test set of Stacking-SVR; (**c**) training set of Poly-SVR; (**d**) test set of Poly-SVR.

Figure 13 presents the relative error frequency distribution of the Poly-SVR model and its optimized counterpart. For the Poly-SVR model, the proportions of samples with relative errors within 5% are 80.87% and 74.14% for the training and test sets respectively. After optimization, these proportions increase markedly to 98.7% and 94.83% respectively. This comparison demonstrates that the Stacking-SVR model achieves significantly higher prediction accuracy than the Poly-SVR model. Therefore, optimizing the SVR model with the Stacking ensemble algorithm proves to be a highly effective approach.

#### 3.3.2. Performance Comparison of Stacking-SVR and Other Models

Table 5 summarizes the predictive performance of the Stacking-SVR model in comparison with Arrhenius, XGBoost, and MLP models on both the training and test sets [50]. These results demonstrate that the Stacking-SVR model consistently outperforms other models across all metrics. Stacking-SVR achieved the lowest *RMSE* value on both the training set and test set, indicating that its fitting accuracy is better than those of Arrhenius (with a value of 13.9778 on the test set), XGBoost (5.4737), and MLP (47.4629). At the same time, Stacking-SVR also has the smallest *MAPE*, with 0.011 for the training set and 0.0169 for the test set, further confirming the model’s precise prediction capability. The coefficient of determination for Stacking-SVR reaches 0.9985 and 0.9982 for the training and test sets, respectively, highlighting its excellent generalization ability. Therefore, these comparisons indicate that the Stacking ensemble strategy effectively combines the strengths of multiple base learners, yielding higher predictive accuracy and stability than individual gradient boosting methods or standard deep learning models. This result justifies the choice of Stacking-SVR as the primary predictive model for the hot deformation behavior of TC18 alloy.

## 4. Conclusions

Based on the hot compression stress–strain curves of TC18 alloy, this study establishes an SVR machine learning model to describe its nonlinear flow behavior, along with a Stacking-SVR optimization model. The effects of different kernel functions, model hyperparameters, and data scales on the fitting and prediction capabilities of the models are systematically investigated. The model evaluation yields the following main conclusions:

(1) The flow stress behavior of TC18 alloy during hot deformation exhibits significant DRX characteristics, with the stress first rising to a peak and then decreasing as the strain increases. Furthermore, both the flow stress and the peak stress show a significant negative correlation with the deformation temperature and a significant positive correlation with the strain rate. This pattern provides a key mechanical basis for optimizing the hot working process of the alloy and controlling its forming quality.
(2) Four SVR models with different kernel functions (linear, polynomial, RBF and Sigmoid) were employed to predict the flow stress of TC18 alloy. Evaluation using three statistical indicators, *R*^2^, *RMSE*, and *MAPE*, indicates that the SVR model with the polynomial kernel function achieves the best prediction performance. For this model, the *R*^2^, *RMSE*, and *MAPE* values are 0.9973, 3.7746, and 0.0233 on the training set, and 0.9949, 4.6864, and 0.0351 on the test set, respectively. The SVR model with the RBF kernel function ranks second in prediction performance, while the models with the linear and Sigmoid kernel functions exhibit the poorest predictive performance.
(3) With the polynomial kernel function selected for the SVR model, the grid search method combined with five-fold cross-validation is employed to optimize the model hyperparameters. When the hyperparameter combination is set to {*C* = 1000, *coef*0 = 1, *d* = 5, *ε* = 1, *γ* = 1}, the *MSE* of each subset reaches a minimum value of 33.28, indicating that both the fitting effect and predictive ability of the model are optimized.
(4) Data scale significantly influences the fitting performance of the model. Considering both the sufficiency of training data and the reliability of independent test evaluation, the 80/20 training–test split was adopted as a reasonable compromise for model development and validation.
(5) The Stacking-SVR model achieves *RMSE* and *MAPE* values of 2.7882 and 0.011 on the training set, with an *R*^2^ of 0.9973; on the test set, the *RMSE* and *MAPE* are 2.7956 and 0.0169, respectively, with an *R*^2^ of 0.9982. All these indicators are better than those of the Poly-SVR, Arrhenius, XGBoost and MLP. The proportion of samples with relative errors within 5% is higher for the optimized Stacking-SVR model, reaching 94.83% on the test set and as high as 98.7% on the training set. These results demonstrate that the Stacking-optimized model significantly enhances both fitting and generalization capabilities, making it more suitable for predicting the deformation behavior of TC18 alloy under different hot working parameters.

## Figures and Tables

**Table 1:** Chemical composition of TC18 alloy.

Alloying Elements	Al	V	Mo	Cr	Fe	O	C	N	Ti
wt%	5.16	4.96	4.92	1.10	0.98	0.18	0.012	0.0012	Balance

**Table 2:** Common kernel functions.

Kernel Function Type	Mathematical Formula	Scope of Application
Linear	$K\left(x_{i},x_{j}\right)={x_{i}}^{T}\cdot x_{j}$	Linearly separable data
Polynomial	$K\left(x_{i},x_{j}\right)={\left(\gamma {x_{i}}^{T}\cdot x_{j}+c\right)}^{d}$	Nonlinear problems of moderate complexity
RBF	$K\left(x_{i},x_{j}\right)=exp\left(-\gamma {∥x_{i}-x_{j}∥}^{2}\right)$	Highly nonlinear data
Sigmoid	$K\left(x_{i},x_{j}\right)=tanh\left(\gamma {x_{i}}^{T}\cdot x_{j}+c\right)$	Some specific scenarios

**Table 3:** Hyperparameter settings.

Parameter	Symbol	Value Range	Physical Meaning
Polynomial degree	d	[1,2,3,4,5]	Controls nonlinear fitting ability
Kernel coefficient	γ	[0.00001, 0.0001, 0.001, 0.01, 0.1, 1]	Determines “sensitive scale” of similarity measure between samples
Independent term	coef0	[0.01, 0.1, 1]	Adjusts low/high-order term balance
Regularization parameter	C	[0.1, 1, 10, 100, 200, 400, 600, 800, 1000]	Controls error penalty intensity
Insensitive loss parameter	ε	[0.001, 0.01, 0.1, 1]	Determines sensitivity threshold of model to regression errors

**Table 4:** Hyperparameter optimization result (rank top 10).

Parameter					Test_Score						Rank
C	coef0	d	ε	γ	Split 0	Split 1	Split 2	Split 3	Split 4	Mean	
1000	1	5	1	1	35.71	41.38	28.26	22.36	38.66	33.28	1
1000	1	5	0.01	1	35.37	53.48	24.63	26.37	37.74	35.52	2
1000	1	5	0.001	1	37.60	50.27	25.81	26.66	37.72	35.61	3
1000	1	5	0.1	1	37.02	53.42	26.01	25.94	39.03	36.28	4
800	1	5	1	1	47.38	47.27	29.17	28.34	45.80	39.59	5
800	1	5	0.01	1	54.56	59.44	29.82	32.42	39.76	43.2	6
800	1	5	0.001	1	53.99	59.45	30.92	32.55	39.81	43.34	7
800	1	5	0.1	1	54.39	59.53	29.69	32.72	40.94	43.46	8
600	1	5	1	1	58.47	53.56	31.06	36.81	49.68	45.92	9
600	1	5	0.1	1	70.28	65.35	31.06	39.43	49.13	51.05	10
…	…	…	…	…	…	…	…	…	…	…	…

**Table 5:** Comparison of different machine learning models.

Model	RMSE		MAPE		R^2^	
	Training Set	Test Set	Training Set	Test Set	Training Set	Test Set
Stacking-SVR	2.7882	2.7956	0.011	0.0169	0.9985	0.9982
Arrhenius	10.3612	13.9778	0.0409	0.0489	0.9785	0.9632
XGBoost	2.9548	5.4737	0.0213	0.0322	0.9983	0.9944
MLP	40.0401	47.4629	0.2828	0.2775	0.6791	0.5756

## Data Availability

The original contributions presented in this study are included in the article. Further inquiries can be directed to the corresponding author.
